# Sugar-Free, Vegan, Furcellaran Gummy Jellies with Plant-Based Triple-Layer Films

**DOI:** 10.3390/ma16196443

**Published:** 2023-09-27

**Authors:** Anna Stępień, Joanna Tkaczewska, Nikola Nowak, Wiktoria Grzebieniarz, Urszula Goik, Daniel Żmudziński, Ewelina Jamróz

**Affiliations:** 1 Department of Engineering and Machinery for Food Industry, Faculty of Food Technology, University of Agriculture, Balicka Street 122, PL-30-149 Cracow, Poland; urszula.goik@urk.edu.pl (U.G.); daniel.zmudzinski@urk.edu.pl (D.Ż.); 2 Department of Animal Products Processing, University of Agriculture, Balicka Street 122, PL-30-149 Cracow, Poland; joanna.tkaczewska@urk.edu.pl; 3 Department of Chemistry, University of Agriculture, Balicka Street 122, PL-30-149 Cracow, Poland; nikola.nowak@urk.edu.pl (N.N.); wiktoria.grzebieniarz@urk.edu.pl (W.G.); 4 Department of Product Packaging, Cracow University of Economics, Rakowicka Street 27, PL-31-510 Cracow, Poland; ewelina.jamroz@urk.edu.pl

**Keywords:** confectionery gels, low-sugar candy, gummy jelly, furcellaran, biodegradable films

## Abstract

Increasing consumer awareness of the impact of nutrition on health and the growing popularity of vegan diets are causing a need to look for new plant-based formulations of standard confectionery products with high energy density and low nutritional value, containing gelatin. Therefore, the aim of this study was to develop vegan and sugar-free gummy jellies based on an algae-derived polysaccharide—furcellaran (FUR). Until now, FUR has not been used as a gel-forming agent despite the fact that its structure-forming properties show high potential in the production of vegan confectionery. The basic formulation of gummy jellies included the addition of soy protein isolate and/or inulin. The final product was characterized regarding its rheological, antioxidant, mechanical and physicochemical properties. Eco-friendly packaging for the jellies composed of a three-layer polymer film has also been developed. It was observed that the highest values of textural parameters were obtained in jellies containing the addition of soy protein isolate, whose positive effect was also found on antioxidant activity. Before drying, all furcellaran-based gel systems showed G’ and G” values characteristic of strong elastic hydrogels. Storing jellies for a week under refrigeration resulted in an increase in hardness, a decrease in moisture content and reduced water activity values. Overall, our study indicates the high potential of furcellaran both as a gelling agent in confectionery products and as a base polymer for their packaging.

## 1. Introduction

Furcellaran is an anionic polysaccharide obtained by the extraction of red algae *Furcellaria lumbricalis*, composed of units consisting of a fragment of (1 → 3) β-d-galactopyranose with a sulfate group at C-4 and (1 → 4)-3,6-anhydro-α-d-galactopyranose, which contains 16–20% sulfate content. Structurally, furcellaran is largely similar to kappa-carrageenan [1,2]. According to European Union legislation, FUR has been approved for use as a food additive functioning as a gel-forming substance and thickener. As interest in algae-derived substances has been growing steadily in recent times, there are reports of explorations for new industrial applications of furcellaran. Studies mainly focus on the use of FUR as a component of biodegradable films to replace synthetic packaging in the food industry [3]. However, due to its gelling properties, the ability to form stable complexes with other polymers and the low energy value resulting from the presence of bonds resistant to human amylases, furcellaran is a promising component that can find an application in the production of confectionary.

*Oenothera biennis* L. as a biennial plant belongs to *Onagraceae* family, which also has many useful biological properties. As a commercial medicinal plant, its oil is used in the pharmaceutical industries, nutraceutical, cosmetic, and feed sectors. It traditionally contains a high content of protein, fiber, vitamin (A and D) and minerals (mainly calcium, potassium and magnesium) [4]. Within the protein composition, it is rich in sulfur amino acids. Oenothera oil is very popular among consumers and is often used as herbal supplements for dietary additions or in the cosmetic and skincare industry. Obtaining oenothera oil generates a lot of protein waste, which can be a valuable raw material with a high nutritional value with active components [5]. So, we have made an assumption that the oenothera by-product obtained after oil extraction, due to its high content of protein as well as complexation/enrichment with polyphenols, can be recommended for use as a promising active ingredient in engineering multi-layer or emulsified films.

The growing interest in a plant-based diet has meant that new vegan alternatives to products containing animal ingredients are constantly appearing on the food market. Therefore, also within the candy segment, new formulations are being searched for to produce jelly beans and gummies without the addition of gelatin [6]. Nevertheless, it is a significant challenge to achieve the resilient structure provided by gelatin using vegan hydrocolloids, so there is a need to find new solutions in this area. Plant-based substitutes for gelatin used as gelling agents in confectionary products include agar [7], carrageenan [6], alginate [8], and pectin [9], among others. However, there are no reports as of yet on the application of furcellaran in this type of food.

Gummy jellies, classified often as so-called confectionery gels containing, among other things, gelling agents, sweeteners and fruit acids, are generally considered to have low nutritional value due to their lack of health-promoting substances and high proportion of simple sugars [10,11]. In addition, the fact that gummies are popular especially among children and adolescents intensifies the need to look for more high-value formulations. Therefore, considering the health of potential confectionary consumers, increasing attention is being given to the development of products that are reduced in calories or incorporate health-promoting additives. As an excessive supply of sugar in the diet contributes to the development of many metabolic diseases [12], one of the directions for modifying gummy jellies is the elimination of sucrose or sugar syrups. For example, confectionery products with sorbitol [13], sucralose and erythritol [14], mannitol [15] and xylitol [16] were reported. Increasing the value of gummy candies can be carried out by adding various types of bioactive substances such as probiotics [17,18], vitamins [9,19] or plant extract [20,21].

Since gummy jellies are a product categorized as a ready-to-go snack, they are sold in small unit packs made of synthetic films, which are categorized as single-use plastic (SUP). It is assumed that synthetic packaging materials represent the highest share of waste classified as SUP [22]. Therefore, the main task implemented as part of rational waste management is the search for alternative biodegradable packaging plastics. One of the most promising solutions, within environmentally friendly packaging technologies, is the deployment of biopolymers. Incorporating waste from food processing into biopolymer films can effectively reduce waste and promote sustainability in the food industry. However, the selection of materials and components for biopolymer films should consider desired properties and the type of food product being packaged. While some biopolymers have shown promise for food packaging, their limitations in physicochemical properties have restricted their application [23]. To overcome these limitations, researchers have explored the combination of various biopolymers, such as proteins, lipids and polysaccharides, in the development of reinforced biopolymer-based films with improved mechanical properties and biological activity [24].

Proteins and polysaccharides are commonly used as feedstock for biopolymer films [25]. It was found that furcellaran, used as a gelling agent in our study, can be also used as a matrix for many biopolymer films due to its ability to form film form [26,27,28]. The incorporation of biopolymer films into extracts with antioxidant or antimicrobial activity can lead to active packaging, extending product quality.

The objectives of this research include the following: (1) to develop a formulation of furcellaran-based sugar-free jellies and to characterize their basic physicochemical properties, (2) to develop technology to produce biodegradable film as packaging for jellies; and (3) to determine the effect of packaging on the basic physical and mechanical characteristics of the product during storage. We aimed to develop a basic gummy formulation that takes into account an FUR, acidity regulator, sweetener, natural color and flavor, and two potential structuring compounds: inulin and soy protein isolate (SPI). Gummy jellies in three different variants were characterized with respect to their textural, rheological and antioxidant properties. Their nutritional value was also estimated, and storage analyses were performed to check the changing of basic qualities over time. Furcellaran was also used to produce a three-layer film enriched with oenothera oil, which was the packaging for jellies. The physical characteristics of gummy jellies stored for a week in polymer films and without packaging were compared. Therefore, the research resulted in an innovative product with packaging, using the same biopolymer—furcellaran as a gelling agent and component of biodegradable films.

## 2. Materials and Methods

### 2.1. Materials

Oenothera by-products were obtained from a local processor Olinii (Nowa Wieś, Poland). This was waste from the production of oenothera cold-pressed oil. To isolate the protein from oenothera, hexane (Sigma Aldrich, St. Louis, MO, USA), hydrochloric acid and sodium (Chemland, Stargard, Poland) were used. The following were employed to make the gummy jellies and films: spirulina powder (Green Essence, Pyrzyce, Poland), furcellaran type 7000 (Est-Agar AS, Karla Village, Estonia), soy protein isolate (Bene Vobis, Gdańsk, Poland), inulin HPX (Beneo Orafti^®^, Mannheim, Germany), citric acid (Biomus, Lublin Poland), cold-pressed oenothera (*Oenothera biennis*) oil from (Oleofarm, Wrocław, Poland), glycerol and Tween 80 (Chemland, Stargard, Poland).

### 2.2. Preparations of Furcellaran Gummy Jellies

The four variants of gummy jellies were prepared according to the formulation shown in Table 1. The weighed amount of furcellaran powders was mixed with distilled water at room temperature and stirred using a magnetic stirrer with simultaneous heating set at 70 °C and 500 rpm. Once the polysaccharide was completely dissolved, the rest of the ingredients were added with the exception of spirulina and flavor, which was dissolved in 10 mL of distilled water and inserted into the solution as soon as it had cooled to 60 °C. The solutions were poured into hexagonal prism molds and left to solidify in the refrigerator for 24 h. Individual gels removed from the molds were spread evenly on perforated trays and dried by convection at 45 °C for 4 h, rotating after half the drying time. For the purposes of further analysis, samples after gelling but before drying will be referred to as gels. Meanwhile, jellies/gummies/candies are the dried samples which are final products.

### 2.3. Rheological Characteristics of the Gels

The rheological properties of the furcellaran sample at the temperatures 6 °C, 20 °C and 40 °C were tested. Rheological tests were performed using an RS-6000 rotational rheometer (ThermoFischer, Karlsruhe, Germany), cone-plate system with an angle of 2°, 35 mm diameter and gap of 1.4 mm. Measurements in the range of linear viscoelasticity (LVE) are the standard criterion when selecting the value of the vibration amplitude γ. The measurements are carried out at a constant frequency value, determining the relationship between the storage modulus G′ and the loss modulus G″ as a function of the strain amplitude. The area of experimental curves is independent of the applied strain amplitude and defines the range of linear viscoelasticity. The appropriate value determination usually consists of determining the maximum amplitude in the LVE range, which translates into the largest possible deformation, and thus increases the accuracy of the measurements [29]. Determination of the relationship between the storage modulus—G′ (Pa) and the loss modulus—G″ (Pa) at a constant frequency of 1 Hz determines the range of linear viscoelasticity. A frequency sweep test, for frequencies from 0.1 to 50 Hz in the range of linear viscoelasticity was carried out. To determine the strength of the gels, the tan(δ) phase angle tangent values as the ratio G″/G′ were calculated.

### 2.4. Physicochemical Properties and Nutritional Labeling of Gummy Jellies

The moisture content of the samples at each stage of the technological process (after gelling, drying and storage) was determined by the gravimetric method in a laboratory dryer at 60 °C until a constant weight was obtained. The water activity was measured in triplicate using AquaLAB 4TE equipment (Decagon Devices Inc., Pullman, WA, USA). The pH values of the gummy jelly solutions were determined using a pH-meter CP-505 (Elmetron, Zabrze, Poland). The values of basic macronutrients of the four variants of gummy jellies were calculated using Cronometer (Revelstoke, BC, Canada) software (https://cronometer.com/, accessed on 20 September 2023).

### 2.5. Antioxidant Activity

The jelly extracts (20 mg/mL) were prepared by adding jelly in the amount of 0.2 g to 10 mL of distilled water. The tubes containing the jelly extracts were put in a water bath at a temperature of 50 °C with shaking action for 10 min to ensure complete dissolution of the jelly. The extracts prepared in such a manner were then used for Ferric Reducing Antioxidant Power Assay (FRAP) and metal-chelating activity.

The FRAP assay was completed according to the method of Benzie and Strain [30]. The FRAP reagent comprised an acetate buffer (pH 3.6), a ferric chloride solution (20 mM), and a 2,4,6-tripyridyl-s-triazine solution (10 mM TPTZ in 40 mM HCl) at the ratio of 10:1:1 (*v*/*v*/*v*), respectively. Firstly, the FRAP reagent was incubated at a temperature of 37 °C in the dark for 30 min, and then, it was mixed with a jelly extract in the ratio of 0.4:3.6 (*v*/*v*). The solution was incubated one more time at 37 °C for 10 min in dark conditions, and the following was absorbance measurement at 593 nm via the Helios Gamma UV-1601 spectrophotometer (Thermo Fisher Scientific, Waltham, MA, USA). The metal-chelating ability of jelly extract was assessed according to the method described by Tkaczewska, Zając, Jamróz, and Derbew [31]. The analyses were performed in duplicate for 3 samples of the jelly (n = 2 × 3).

### 2.6. Texture Profile Analysis

The texture properties of gels and gummy jellies were evaluated by a two-bite compression test using a Shimadzu EZ Test EZ-LX (Shimadzu, Kyoto, Japan) universal testing machine equipped with a load cell of 500 N. Before analyses, jellies were rested at room temperature 30 min before the test. Each sample was compressed twice to 50% of its original height with a 36 mm diameter cylindrical press jig, which allowed the jelly to deform without penetration. The test speed was 1 mm/s, and the trigger point was 0.05 N. The obtained reports for this test included hardness, springiness and cohesiveness. Textural parameters values were determined using Trapezium X software (https://www.shimadzu.eu/trapezium-x-software, accessed on 20 September 2023) (Shimadzu, Kyoto, Japan). Analyses were carried out in 10 replicates.

### 2.7. Film Preparations, Packing and Storage Analysis

#### 2.7.1. Isolation of Protein from Post-Production Oenothera Pomace

Isolation of the protein was performed according to Trigui et al. [32]. Oenothera pomace was ground using a laboratory mill. It was then defatted by placing them in hexane ((1:5 *w*/*v*), pomace: hexane) for 4 h. The procedure was repeated twice. The defatted pomace was placed under a fume hood for 24 h at room temperature to evaporate the hexane. The as-prepared pomace was dispersed in distilled water and raised to pH 11 using a previously prepared 1.0 M NaOH solution. The pH value was controlled continuously using a CP-505 pH-meter (Elmetron, Zabrze, Poland). The suspension was stirred on a magnetic stirrer (MR Hei-Tec, Heidolph Instruments GmbH & Co. KG, Schwabach, Bayern) for 2.5 h at 30 °C. After this time, the suspension was centrifuged 3000× *g* at 25 °C for 20 min, and the supernatant was collected. The process was repeated twice, and all supernatants thus obtained were combined. Subsequently, a pH value of 4.5 was set to precipitate the protein using a previously prepared 1 M HCl solution. The solutions were centrifuged again, the supernatant was discarded and the precipitate was washed with water, lyophilized and stored at −20 °C until analysis.

#### 2.7.2. Preparation of the Emulsion and Spirulina Solutions

About 2 g of protein isolated from oenothera pomace was dissolved in buffer pH 11. Then, 5 mL of oenothera oil and 5 mL of Tween 80 were added. The mixture was homogenized using a Polytron PT2500E homogenizer (Dan-Lab, Białystok, Poland). Aqueous spirulina solution was prepared by dissolving 2 g of spirulina in 80 g of water. It was then stirred using a magnetic stirrer for 30 min (400 rpm, temp. 30 °C).

#### 2.7.3. Preparation of the Multilayer Film

**Figure 1:**
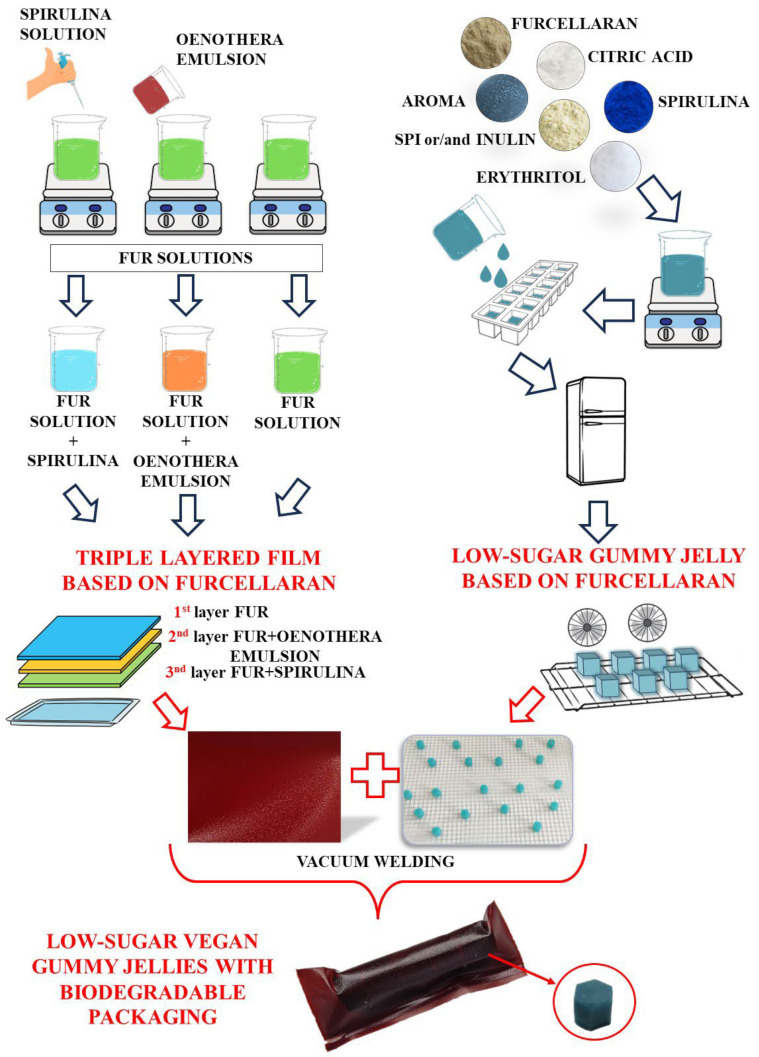
Production scheme for furcellaran-based jelly and packaging films.

A solution of 1% furcellaran was prepared and glycerol (1% *w*/*w*) was added as a plasticizer. The so-obtained solution was stirred under continuous heating (temperature 100 °C) to dissolve the polymer. Then, the temperature of the solution was lowered to 60 °C, and 250 mL was taken and poured onto a previously prepared form to turn into a gel. This formed the 1st layer of the biopolymer film. Then, 200 mL of the solution was taken and 50 mL of the previously prepared emulsion was added to it, which constituted the 2nd layer of the biopolymer film. After thorough mixing, the solution was poured over the gelled 1st layer and again left until it turned into a gel. The 3rd layer was made up of 240 mL of furcellaran solution and 10 mL of spirulina aqueous solution. After spilling the third layer, the composite was left to dry under a laboratory fume hood at room temperature. The procedure for obtaining three-layer films and their active ingredients is shown in Figure 1.

#### 2.7.4. Packing and Storage

After drying, the gummy jellies were cooled to room temperature, piled one on top of the other in 10-piece packets, and wrapped in biopolymer film, which was then sealed in tube form using a food vacuum sealer Freshpack Pro-QH(QH-01) (SaengQ, Wenzhou, China). Gummies packaged in film, and jellies without packaging as reference samples, were stored for 7 days in a climate chamber KBK-140W (Wamed, Warsaw, Poland) at 25 °C under 60% relative humidity. After one week, the samples were removed from the film, weighed and examined for textural properties and water activity according to the methodology described in earlier paragraphs.

### 2.8. Statistical Analyses

Averages and standard deviations of samples were reported based on the triplicate measurement. Statistica (StatSoft, Inc., Tulsa, OK, USA) software version 13.3 was used to perform multifactor Analyses of Variance (ANOVAs) in order to determine whether the effect of the variables (formulation and stage of the technological process) on the product was significant. For the other variables, significant differences (*p* < 0.05) were evaluated using one-way ANOVA with the Duncan test.

## 3. Results and Discussion

### 3.1. Rheological Characteristic of Furcellaran Gel Systems

The rheological characteristics of furcellaran gels allow studying the physical stability, esthetic effect, quality and usability of obtained preparations. The rheological studies allow researchers to understand and evaluate the response of a material to various inputs, such as stress or strain at different frequencies and at different temperatures. Results of rheological analyses are also useful for evaluating product consistency as well as the effect of the additives on the product itself. The rheological properties of hydrogels allow them to be classified as viscoelastic materials because they exhibit both elastic and viscous behavior and exhibit physical properties between liquid and solid states. The frequency sweep test allows researchers to observe how the relationship between the viscous and elastic moduli as a function of frequency was changed. The frequency sweep test is performed within the linear viscoelastic region (LVER), which is defined by the initial constant frequency amplitude sweep test to avoid sample damage. In this range, G′, G″ and tan (δ) do not change with the applied strain [33].

**Figure 2:**
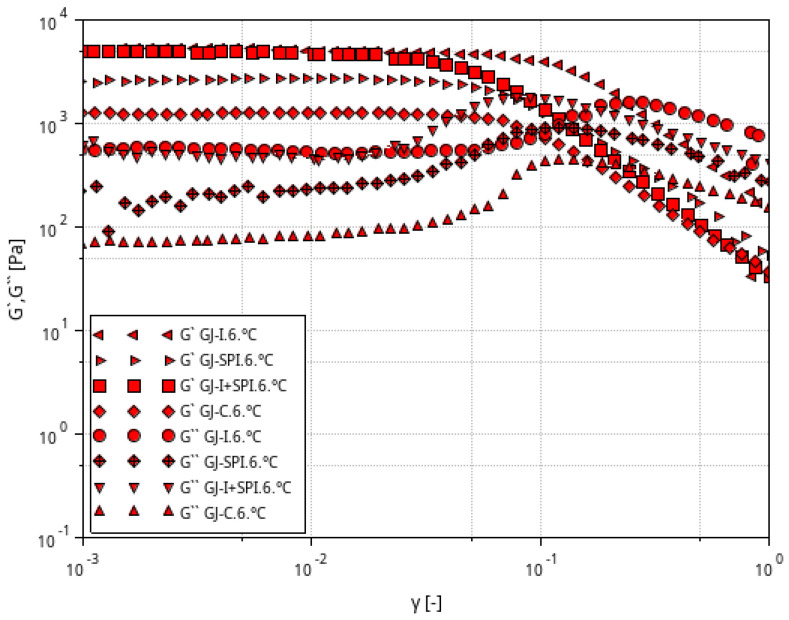
Relationship between the G′ and G″ modulus values as a function of strain amplitude at constant oscillation frequency for furcellaran-based gels at a temperature of 6 °C.

**Figure 3:**
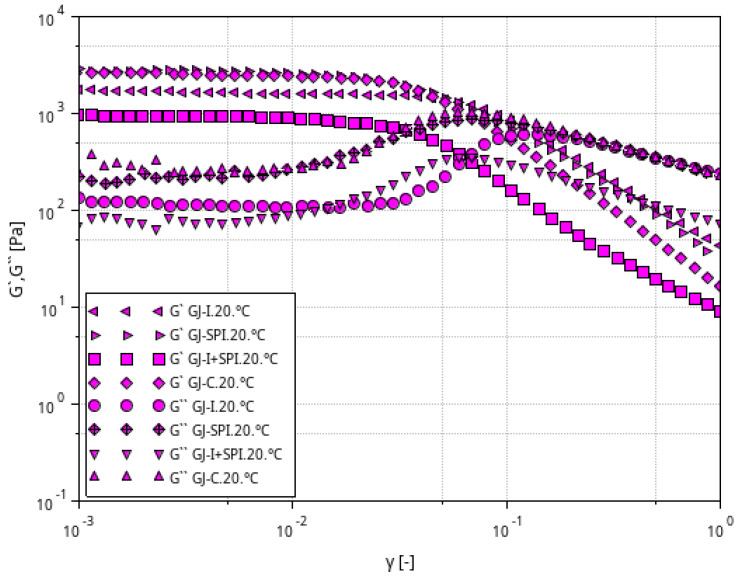
Relationship between the G′ and G″ modulus values as a function of strain amplitude at constant oscillation frequency for furcellaran-based gels at a temperature of 20 °C.

**Figure 4:**
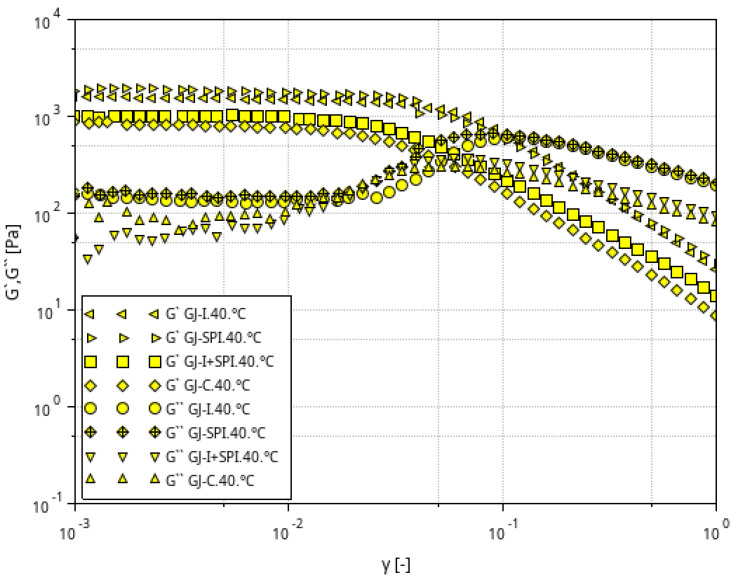
Relationship between the G′ and G″ modulus values as a function of strain amplitude at constant oscillation frequency for furcellaran-based gels at a temperature of 40 °C.

Figure 2, Figure 3 and Figure 4 shows the storage modulus G′ and the loss modulus G″ as a function of strain for the tested furcellaran-based gels. The tested samples did not show quantitative similarity. At low strain amplitudes, G′ was almost constant, which suggest a linear viscoelastic range in which there is no permanent damage to the sample structure. Then, both modules become strain dependent, with G′ decreasing and G″ passing through the peak which is located just before or at the intersection point whose strain is defined as destructive [33]. A similar behavior of the samples was observed in different systems where bacterial alginate-like exopolymers (ALE) gels were investigated [34,35].

**Figure 5:**
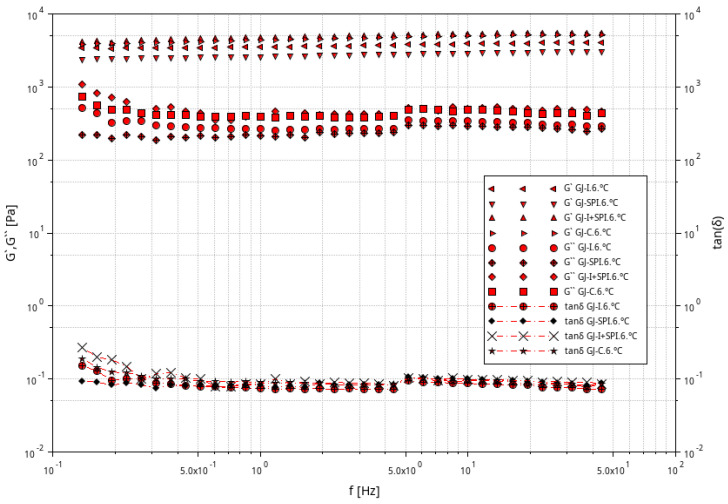
Dependence of G′ and G″ modulus on frequency for furcellaran-based gels at 6 °C and the dependence of the tan (δ) phase shift angle tangent on frequency for examined systems.

**Figure 6:**
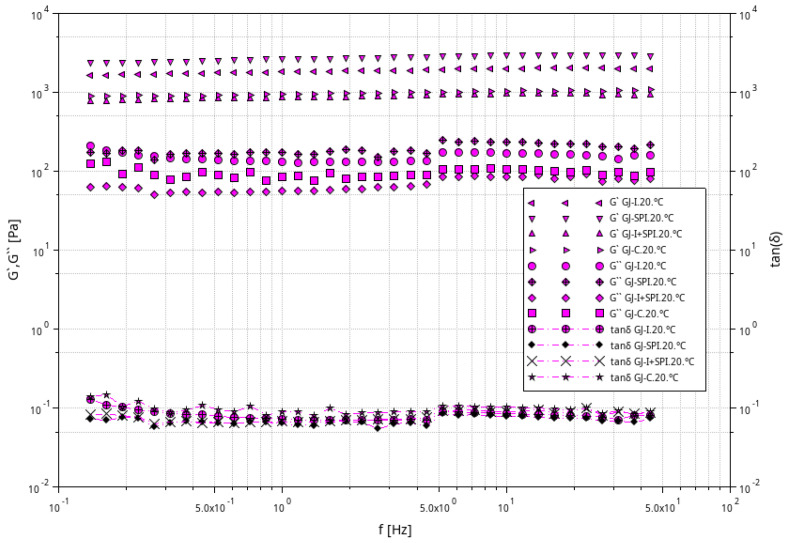
Dependence of G′ and G″ modulus on frequency for furcellaran gels at the temperature of 20 °C and the dependence of the tan (δ) phase shift angle tangent on frequency for examined systems.

**Figure 7:**
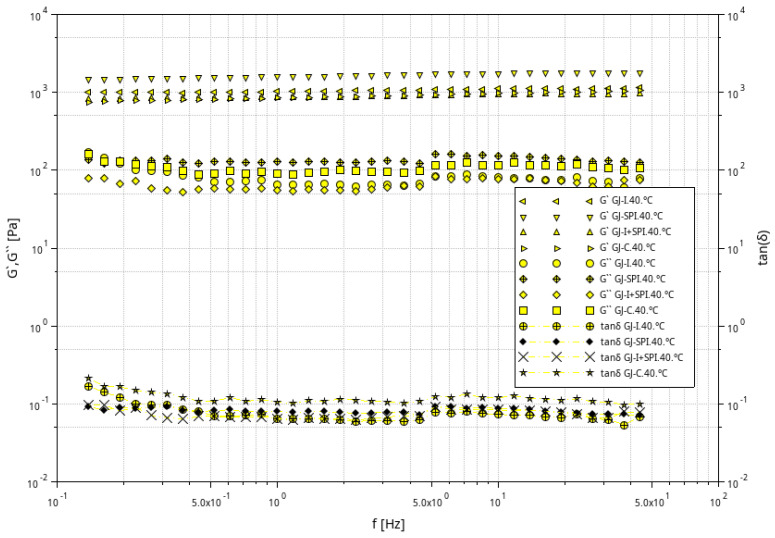
Dependence of G′ and G″ modulus on frequency for furcellaran-based gels at the temperature of 40 °C and the dependence of the tan (δ) phase shift angle tangent on frequency for examined systems.

The results of the frequency sweep test obtained at temperatures of 6 °C, 20 °C and 40 °C are presented graphically in Figure 5, Figure 6 and Figure 7. For all samples, G′ is always about 10 times greater than G′′ at the full frequency range at all tested temperatures 6 °C, 20 °C and 40 °C. G′ and G′′ run almost parallel without an intersection point, which indicates the elastic nature of the cross-linked gels prevailing over the viscous nature in the tested conditions. This behavior is a typical feature of a strong gel system, which suggest strong gel formation for all furcellaran hydrogels; similar behavior was reported by Yang et al. [36].

The response of the elasticity modulus is independent of the frequency in the range from 0.1 to 50 Hz. The G′′ moduli were almost frequency-independent in the range of 0.3–50 Hz, with the exception of samples GJ-I, GJ-SPI and GJ-C at 60 °C, where a clear decrease in G″ in the range of 0.1–0.3 Hz was observed. A characteristic slight increase in G″ at the frequency of 5 Hz was also observed for all tested systems. Similar behavior of the G′ and G′′ modules was reported for 2% kappa-carrageenan gels [37]. The lack of literature about the rheology of furcellaran-based gels encourages further research on these systems. The ratio of the lost energy to the stored energy in each cycle is described as tan(δ), which informs about the physical behavior of the analyzed material. The phase angle tangent (tan(δ) = G″/G′) showed the relative importance of the viscosity modulus G″ and the elastic modulus G′ [38]. The obtained values of tan(δ) were always lower than one, which confirms the constant behavior of the gels at all range of the frequency values and confirms a high degree of cross-linking of the gels. At lower frequency values of ~0.1–1 Hz, the examined systems have a tan(δ) value between 0.3 and 0.1 at 6 °C and 20 °C, while at the higher frequencies, tan(δ) values are below 0.1. All the evaluated furcellaran-based gels showed a strong gel network in the entire tested frequency and temperature range [39]. However, at the temperature of 40 °C, the control (GJ-C) gel has values higher than 0.1 in the entire frequency range, which causes the weakening of the gel network. Therefore, it can be concluded that the tested systems were strong gels, and the additions of inulin and soy protein isolate did not change the nature of the strength of the gels; it can even be assumed that they keep it constant. Analyzing the evaluated systems, it can also be seen that the GJ-SPI sample with the addition of SPI was the most stable, because the values of the G′ and G″ modules are constant over the entire range of tested frequencies and temperatures.

### 3.2. Physicochemical Properties and Nutritional Labeling of Gummy Candies

Table 2 shows basic physicochemical data: sample weights, water activity and pH of low-sugar jellies during the following manufacturing stages. The experiments included four sample formulations and characteristics: gels before drying, jellies after drying, jellies stored in film (obtained as described in Section 2.7.1) and jellies stored without packaging. The weight of the samples after cooling and gelling varied between 9.54 g for the GJ-SPI sample and 10.03 g obtained for the variant with the addition of 2% inulin. These differences are caused by different formulations. However, except for the control sample, all versions were characterized by the same dry matter content; thus, these differences turned out to be statistically insignificant. The transformation of gels into gummy jellies includes drying in which excess water is removed, thus changing the structural properties of the product and stabilizing its structure. Therefore, the selection of proper process conditions including time and temperature is essential for the quality of the final product. During the production of commercial gummy candies, water is removed so as to ensure not only their high quality but also to provide optimum efficiency and continuity of production while taking into account the energy requirements important for economic reasons. For all variants of furcellaran-based gel systems, approximately twofold weight loss was observed during convection drying. It is assumed that the standard moisture content of jellies varied from 8 to 22% [11]. In our study, drying was carried out for four hours at a relatively low temperature so as to possibly preserve all thermolabile components such as volatile aromatic compounds as a result of which the final jellies had a significantly higher (64–66%) than recommended moisture content. The choice of such a method for removing moisture from furcellaran gels was predicated on the fact that priority was given to evaluating the macroscopic appearance of the sample during drying, especially homogeneous surface without signs of crystallization for erythritol.

In order to test the effect of biopolymer packaging on the properties of jellies, they were stored under refrigeration (temperature: 5 °C, relative humidity: 60%) for one week. The reference sample consisted of candies stored without packaging. As a result of controlling the weight of jellies after storage, we observed a decrease in the average weight for both wrapped and unwrapped samples. As a result of controlling the weight of jellybeans after storage, we observed a decrease in the average weight for both wrapped and unwrapped samples with more water lost by the unpackaged samples. Thereby, it can be concluded that the water content of the material was too high to store it in an unchanged state under the refrigeration conditions. The average weight of jellies unit after 7 days of packaged candies varied from 3.83 to 4.38 g, while for unpackaged candies, the values were in the range of 3.15–3.63 g. The greatest weight loss after storage (about 18%) was observed for samples containing inulin. This means that furcellaran alone binds and cross-links water better in gel systems than furcellaran with added inulin. The negative effect of inulin on the structure of furcellaran gels may also be due to its tendency toward crystallization, which is determined by the polymer chain length and its initial concentration in the system [40]. Water loss in jellies stored in triple-layer films ranged from 1.2 to 5.2%, indicating that despite the sealed packaging, free water migrated from the product structure into the film or optionally to the environment. Since, depending on the formulation, the decrease in moisture content during sample storage varied, it can be concluded that the physical stability of investigated furcellaran-based gel systems primarily depends on their composition and the solids–water interaction.

**Figure 8:**
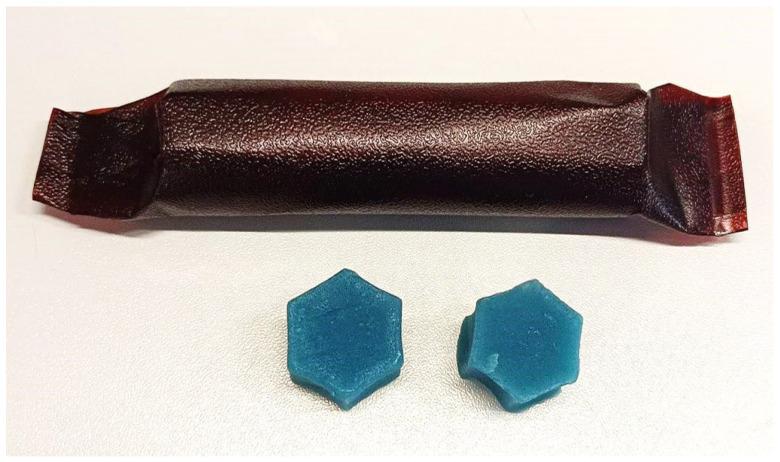
Example photo of the gels and the final packaging on the product.

An attempt was made to produce a multi-layer packaging based on furcellaran, which was enriched with oenothera emulsion and spirulina. The packaging was designed to meet the needs of consumers who are environmentally conscious and want to reduce synthetic waste from food packaging. The material designed in this way was intended to contribute to extending the shelf life of gummy jellies. The appearance of a portion of the jellies in a biopolymer package is shown in Figure 8.

Despite the fact that moisture level has a significant influence on texture and shelf life, water content by itself is not sufficient to completely characterize gummy jelly quality and stability during storage. A parameter often used to describe microbial stability, texture and water migration over time is water activity defined as the ratio of the vapor pressure of the material to the vapor pressure of pure water [11]. From a practical point of view, the a_w_ value indicates the amount of water not associated with other components in the structure of the material. This type of water is considered available for various chemical, physical, enzymatic or microbiological processes that in food cause deteriorative reaction during storage. In our study, water activity after drying decreased about 0.15 and ranged from 0.819 to 0.843, which is relatively high compared to literature reports. Gok et al. [15] obtained an a_w_ value that ranged between 0.76 and 0.84 for low-calorie jellies with mannitol addition, while Periche et al. [41] determined an a_w_ level from 0.721 to 0.908 for gummies based on isomaltulose. The high a_w_ values obtained for furcellaran jellies are due to insufficient moisture removal during drying. It is believed that the safe-for-consumers water activity value of this type of range should be between 0.55 and 0.75 [11]. Hence, in order to improve the formulation of the proposed product, it would be necessary to consider changing the drying parameters or increasing the content of solutes, especially those that tend to bind water. It was observed that after 7 days of storage, the water activity of all variants of the gels decreased slightly with a slightly greater decrease for the packaged samples. This tendency can be explained by the fact that every physical system tends toward thermodynamic equilibrium. Since the water activity of the gels was high, non-bonded moisture migrated. The phenomenon was more intense for samples in film with an a_w_ equal to 0.433 than for gummies without package stored in an environment with relative humidity 60% (a_w_ = 0.6).

The main goal of this work was to design vegan gummies, while the secondary goal was to develop a packaging that would be an alternative to synthetic packaging. Multi-layer films based on furcellaran are characterized by improved functional properties in relation to single-layer films, which is certainly due to their complex structure [26,42,43]. The addition of spirulina and oenothera emulsion to a specific layer was intended to give the films barrier properties.

Acidity (pH) is an important factor affecting the safety and quality of food products. As expected, drying the gels to make the final product resulted in an increase in pH. The production of standard confectionary products usually includes the addition of fruit acids such as citric and tartaric. The purpose is to lower the pH value but also to give the product its characteristic sour taste. As expected, the original acidity (3.94–4.24) of furcellaran gels decreased (3.29–3.71) as a result of drying. Acidity is a resultant of material composition; therefore, depending on the applied ingredients, it varies significantly even within a product of the same type. Lekahena and Boboleha [13] obtained seaweed jellies with pH between 4.64 and 4.74, while the acidity of gelatin gummies developed by Delgado and Banon [44] was equal to 3.07.

Standard commercially available gummy jellies are mostly characterized by low nutritional value and high energy density, making them widely considered an “unhealthy consumer choice”. Depending on the type and manufacturer, the energy value of gummies can range from 300 to 400 kcal in 100 g of product. Thus, assuming an average daily caloric intake of 2000 kcal, the consumption of the entire package can correspond to as much as one-fifth of the daily energy requirements. The energetic value of furcellaran-based jellies ranged from 29.3 to 37.3 kcal/100 g (Table 3). Assuming that, according to the results described above, the developing formulation requires the removal of more water, the calculated caloric value of the developed product is low. Potentially reducing the moisture content of the product, even by half, will still result in an energy value of less than 100 kcal/100 g. The highest protein content at the level of 4.07 g was, of course, shown by the sample with the addition of SPI, while in order to increase the value of the product in future research, it is planned to increase the addition of soy protein isolate. Dietary fiber ranging from 5.50 to 8.7 g/100 g could also be higher due to its beneficial effects on health. As expected, the gummies had a low fat content. The carbohydrates, resulting mainly from the erythritol and furcellaran presence, ranged from 28.66 to 30.27 g/100 g, while according to the assumptions, the product did not contain any simple sugars at all. As stated by regulation (EU) 1924/2006 [45] on health and nutrition, sugar-free claims may only be used if the product contains no more than 0.5 g of sugar per 100 g. The gummy jellies developed in this study therefore can be included into this group of food products.

### 3.3. Antioxidant Activity

Table 4 shows the results of investigating the antioxidant activity of furcellaran-based samples at two different stages of the production process. The highest antioxidant activity, expressed both as Ferric Reducing Antioxidant Power and metal-chelating activity, is found in freshly prepared jellies containing soy protein isolate (0.68 uM Trolox equivalent/mg and 20.19%, respectively). As the SPI content of the jellies decreases, their antioxidant activity also decreases. Soy protein isolates (SPIs) are a highly refined form of protein derived from soybeans. They are commonly used in various food products, including beverages, protein bars, meat alternatives, and baked goods. One of the notable properties of soy protein isolates is their antioxidant activity, which can have several health benefits [46]. According to literature data [47], soy protein isolates commonly contain a lot of phenolic compounds, including isoflavonoids that could stabilize free radicals. Both the native soy protein structure and also possibly the presence of residual antioxidant phenolic compounds could cause an antioxidant effect. Furthermore, nonhydrolyzed SPI has been reported to be able to inhibit lipid oxidation and retard rancidity odor development in various model products [48,49].

No changes in the antioxidant activity of the jelly were noted during the 7-day storage. There was also no negative effect of packaging the jelly in biopolymer films on their antioxidant properties. Polyphenols themselves can undergo oxidation when exposed to oxygen in the air. This process can lead to a decrease in their antioxidant capacity. The loss of antioxidant activity due to oxidation can impact the overall effectiveness of the polyphenol-rich product in neutralizing free radicals [50]. Therefore, the lack of change in the antioxidant activity of stored samples is a positive development. However, it would be necessary to conduct further studies in which the storage time of the samples is extended.

The impact of biopolymer packaging on the active properties of products stored within them can vary depending on several factors such as type of biopolymer used, the nature of the product, and storage conditions [24]. Proper selection of biopolymer materials, design considerations, and testing under relevant storage conditions are essential to ensure that the packaging maintains or enhances the active properties of the products it contains.

### 3.4. Textural Properties of Gummy Jelly

The textural properties of food products classified as confectionery have a major impact on consumer perception and acceptance. Our study applied an instrumental TPA technique in order to determine the hardness (N), springiness, cohesiveness and gumminess (N) of furcellaran low-sugar jellies with inulin and/or soy protein isolate additions. The obtained results and statistical effect of the formulations and technological process stage are shown in Table 5.

Four different stages of jelly production were considered in the study: after gelling, after drying, and after one week of refrigerated storage in bioactive film or without packaging. The most significant change in all examined parameters, independently of the sample composition, was observed after drying the gels, which is clearly related to the significant loss of water. In addition, jellies stored in active furcellaran film for seven days showed higher values of hardness, cohesiveness, gumminess and springiness than analogous samples stored in the refrigerator without packaging. This trend is also related to differences in moisture content. As shown in the previous paragraph, the water activity of the storage environment was 0.55, while the a_w_ value of furcellaran films was equal to 0.43. Therefore, jellies hermetically sealed in film, according to the base of thermodynamic equilibrium, gave up more moisture than samples without packaging. There are almost no reports in the literature on the textural properties of confectionary gels based on furcellaran. In addition, it is worth noting that the values of individual parameters determined by TPA depend on the geometry of the tested sample, so it is necessary to take into account not only its composition but also its shape and size [10]. Regardless of the stage of production, hardness values, defined as the maximum force required for the sample deformation in the first compression, were the highest for jellies with 2% soy protein isolate additions and ranged from 8.264 N determined for the sample after gelling to 33.542 N obtained for packing jellies. The increased hardness of the system as a result of the addition of proteins is due to their structure-forming properties. There are no reports on the properties of composite hydrogel systems containing furcellaran and protein, while the increase in hardness of gels based on carrageenan and soy protein has been proven [51]. However, interestingly, both furcellaran and SPI chains are negatively charged, so the thermodynamic incompatibility between the protein and polysaccharide fractions had a positive effect on the textural properties of the samples [52]. Slightly lower hardness than the GJ-SPI samples, at all stages of production, were found for the jellies with the addition of both protein isolate and inulin. Meanwhile, the values determined for the GJ-I+SPI systems are more similar to those obtained for gels with the addition of protein alone. This shows that furcellaran–SPI interactions are definitely stronger than those between inulin and protein. Compatibility between protein–polysaccharide systems, which determines good mechanical properties, is a result of many factors including their type, composition, molecular weight, structure and chain branching [10]. Obtained values of cohesiveness, expressed as internal bonds in the sample and springiness connected with elastic recovery that occurs in the material after compressive force removal, showed the same tendency regarding the hardness. This therefore confirms the key effect of the addition of the protein fraction on the mechanical characteristics desired in confectionary products. Gumminess is a measure representing the energy required to break down a semi-solid product ready for swallowing. The highest values of this texture variable were determined at each stage of the production process for gels containing soy protein isolate, while gumminess is far more determined by the stage of the production process than by the formulation. It was noted that for all systems tested except the GJ-C control sample, drying the gels resulted in a more than threefold increase in gumminess values. It has been demonstrated that the increase in gumminess values as a result of drying can be explained by both gelation and dehydration phenomena. The simultaneous actions of both gelling agents on the colloidal system containing water and other food components resulted in the typical firm and chewy structure of gummy candies [44].

Statistical analysis (multi-factor ANOVA) shows that the technological process step was the variable with the most influence (the highest values of F-ratio) on every textural parameter with the most significant impact on gumminess. The formulation of the jellies exhibited a remarkable effect on the obtained values of cohesiveness and gumminess. A statistically significant effect of both individual variables, process step and formulation, was found for each of the textural parameters, while the F-ratio value for springiness was the highest. The effect of the samples’ formulation on hardness and springiness was not statistically significant.

## 4. Conclusions

The results of the current research indicate that furcellaran has high potential as a gelling agent used in the production of sugar-free gummy jellies. The performed rheological characterization showed that hydrogels based on this polysaccharide are characterized by high stability and elasticity, which confirms furcellaran’s compatibility with other ingredients used in the formulation: erythritol, soy protein isolate and inulin. After drying, the gels transformed into gummies by removing about 50% of the water showed water activity ranging from 0.819 to 0.843 and pH between 3.29 and 3.71. The most significant metal-chelating activity of 29.11% was determined for the variant of jellies with soy protein isolate. The hardness of the dried jellies, which is the most important textural parameter, ranged from 7.31 to 16.38 N, and its value increased during storage regardless of the packaging applied. The use of three-layer furcellaran film as packaging for jellies did not have any negative effect on the antioxidant activity, texture or physicochemical properties of the product. During 7 days of storage, jellies lost only a small amount of water, which is not due to the use of packaging but rather due to the insufficient drying of the product. Therefore, further research will include formulation improvements that take into account an increase in the final dry matter content. Since the use of erythritol as a sweetener did not in any way reduce the mechanical stability or properties of the jellies, future research will be conducted in the direction of increasing the proportion of protein in the product in order to obtain low-sugar confectionery with high nutritional value, which is suitable for consumers who limit sugar in their diets. In order to make the product available at a larger scale, it is also necessary to investigate the effect of biopolymer films on the microbiological safety of jellies under different storage conditions. Nevertheless, based on the results obtained so far, it can be concluded that the use of furcellaran as both a gelling agent and a component of the film makes it possible to obtain a complete vegan confectionary product with environmentally friendly packaging.

## Figures and Tables

**Table 1:** Furcellaran gummy jellies formulation.

Component (g/100 g)	Sample Code			
	GJ-I	GJ-SPI	GJ-I+SPI	GJ-C
Furcellaran	4.00	4.00	4.00	4.00
Erythritol	10.00	10.00	10.00	10.00
Soy protein isolate	0.00	2.00	1.00	0.00
Inulin	2.00	0.00	1.00	0.00
Citric acid	0.20	0.20	0.20	0.20
Spirulina	0.25	0.25	0.25	0.25
Aroma	0.01	0.01	0.01	0.01
Water	83.55	83.55	83.55	85.55

**Table 2:** Physicochemical parameters of the developed low-sugar gummy jellies.

		Weight (g)	Water Activity	pH
after gelling	GJ-I	10.03 ± 0.19 ^f^	0.982 ± 0.001 ^b^	3.94 ± 0.09 ^b^
	GJ-SPI	9.54 ± 0.14 ^f^	0.980 ± 0.001 ^b^	4.24 ± 0.12 ^cd^
	GJ-I+SPI	9.55 ± 0.09 ^f^	0.980 ± 0.002 ^b^	4.02 ± 0.06 ^b^
	GJ-C	9.75 ± 0.20 ^f^	0.981 ± 0.001 ^b^	4.13 ± 0.12 ^cd^
after drying (4 h/45 °C)	GJ-I	4.80 ± 0.20 ^e^	0.819 ± 0.004 ^a^	3.29 ± 0.11 ^a^
	GJ-SPI	4.45 ± 0.18 ^de^	0.837 ± 0.004 ^a^	3.71 ± 0.15 ^e^
	GJ-I+SPI	4.53 ± 0.24 ^de^	0.843 ± 0.006 ^a^	3.34 ± 0.12 ^a^
	GJ-C	4.26 ± 0.26 ^d^	0.842 ± 0.003 ^a^	3.41 ± 0.02 ^a^
after storage without packing	GJ-I	3.15 ± 0.17 ^a^	0.816 ± 0.008 ^a^	
	GJ-SPI	3.63 ± 0.16 ^b^	0.828 ± 0.008 ^a^	
	GJ-I+SPI	3.61 ± 0.21 ^bc^	0.841 ± 0.012 ^a^	
	GJ-C	3.49 ± 0.22 ^bc^	0.837 ± 0.036 ^a^	
after storage with packing	GJ-I	4.17 ± 0.21 ^d^	0.815 ± 0.004 ^a^	
	GJ-SPI	4.22 ± 0.10 ^d^	0.822 ± 0.003 ^a^	
	GJ-I+SPI	4.38 ± 0.31 ^d^	0.837 ± 0.004 ^a^	
	GJ-C	3.83 ± 0.22 ^c^	0.836 ± 0.007 ^a^	

Values with different letters in the same row present significant differences.

**Table 3:** Nutritional values of the developed low-sugar gummy jellies.

	GJ-I (100 g)	GJ-SPI (100 g)	GJ-I+SPI (100 g)	GJ-C (100 g)
Energy (kcal/kJ)	33.0/138.95	33.2/138.95	37.3/156.06	29.3/122.63
Protein (g)	0.21	4.07	2.11	0.23
Total carbohydrate (g)	30.27	27.21	28.66	29.08
Dietary fiber (g)	8.77	5.14	6.95	5.50
Sugars (g)	0.00	0.00	0.00	0.00
Fat (g)	0.00	0.21	0.21	0.00

**Table 4:** Antioxidant activity of the developed low-sugar gummy jellies.

	Sample	FRAP uM Trolox Equivalent/mg	Metal-Chelating Activity (%)
after drying (2 h/35 °C)	GJ-I	0.56 ± 0.04 ^ad^	0 ± 0.00 ^a^
	GJ-SPI	0.86 ± 0.06 ^b^	20.19 ± 2.47 ^c^
	GJ-I+SPI	0.58 ± 0.04 ^abd^	7.51 ± 1.08 ^b^
	GJ-C	0.50 ± 0.10 ^ac^	0 ± 0.00 ^a^
after storage without packing	GJ-I	0.60 ± 0.04 ^abd^	0 ± 0.00 ^a^
	GJ-SPI	0.69 ± 0.05 ^b^	29.11 ± 1.47 ^c^
	GJ-I+SPI	0.60 ± 0.02 ^abd^	8.69 ± 2.03 ^b^
	GJ-C	0.44 ± 0.01 ^c^	0 ± 0.00 ^a^
after storage with packing	GJ-I	0.52 ± 0.11 ^ac^	0 ± 0.00 ^a^
	GJ-SPI	0.66 ± 0.04 ^d^	29.11 ± 5.65 ^c^
	GJ-I+SPI	0.52 ± 0.03 ^ac^	6.34 ± 2.54 ^b^
	GJ-C	0.42 ± 0.05 ^c^	0 ± 0.00 ^a^

Values with different letters in the same row present significant differences.

**Table 5:** Comparison of texture parameters of furcellaran low-sugar gummy jellies.

	After Gelling			
	GJ-I	GJ-SPI	GJ-I+SPI	GJ-C
Hardness (N)	3.582 ± 0.30 ^a^	8.827 ± 0.56 ^c^	8.264 ± 0.57 ^bc^	2.743 ± 0.27 ^a^
Cohesiveness (N)	0.148 ± 0.021 ^ac^	0.346 ± 0.026 ^d^	0.227 ± 0.016 ^bc^	0.139 ± 0.029 ^a^
Springiness	0.079 ± 0.014 ^a^	0.103 ± 0.004 ^a^	0.094 ± 0.006 ^a^	0.058 ± 0.007 ^a^
Gumminess (N)	3.241 ± 0.633 ^a^	5.723 ± 0.278 ^b^	4.424 ± 0.844 ^ab^	3.537 ± 0.191 ^a^
	**After drying [4 h/45 °C]**			
	**GJ-I**	**GJ-SPI**	**GJ-I+SPI**	**GJ-C**
Hardness (N)	8.495 ± 0.81 ^bc^	16.483 ± 0.88 ^e^	15.138 ± 0.81 ^d^	7.313 ± 0.30 ^b^
Cohesiveness (N)	0.251 ± 0.025 ^b^	0.489 ± 0.079 ^e^	0.444 ± 0.054 ^e^	0.198 ± 0.010 ^ab^
Springiness	0.482 ± 0.057 ^c^	0.607 ± 0.040 ^ef^	0.508 ± 0.075 ^cd^	0.236 ± 0.031 ^b^
Gumminess (N)	11.734 ± 0.971 ^d^	16.321 ± 1.010 ^f^	15.681 ± 0.852 ^f^	7.681 ± 0.852 ^c^
	**After 7 days without packing**			
	**GJ-I**	**GJ-SPI**	**GJ-I+SPI**	**GJ-C**
Hardness (N)	16.476 ± 0.84 ^e^	20.680 ± 0.93 ^g^	19.073 ± 1.41 ^f^	15.144 ± 0.82 ^d^
Cohesiveness (N)	0.383 ± 0.012 ^bc^	0.625 ± 0.083 ^f^	0.437 ± 0.029 ^e^	0.286 ± 0.027 ^cd^
Springiness	0.533 ± 0.017 ^cde^	0.626 ± 0.069 ^f^	0.570 ± 0.024 ^def^	0.273 ± 0.019 ^b^
Gumminess (N)	12.863 ± 0.861 ^de^	21.087 ± 0.995 ^h^	19.265 ± 0.935 ^g^	8.392 ± 0.950 ^c^
	**After 7 days with packing**			
	**GJ-I**	**GJ-SPI**	**GJ-I+SPI**	**GJ-C**
Hardness (N)	26.114 ± 0.98 ^g^	33.546 ± 0.60 ^i^	30.566 ± 0.77 ^h^	18.286 ± 1.16 ^f^
Cohesiveness (N)	0.584 ± 0.060 ^f^	0.914 ± 0.051 ^g^	0.850 ± 0.053 ^g^	0.348 ± 0.021 ^d^
Springiness	0.730 ± 0.090 ^g^	0.965 ± 0.089 ^g^	0.825 ± 0.088 ^h^	0.557 ± 0.053 ^cdef^
Gumminess (N)	13.980 ± 0.779 ^e^	28.170 ± 1.275 ^i^	20.642 ± 1.023 ^h^	8.506 ± 0.779 ^c^
	**ANOVA (F-ratio)**			
	**Hardness**	**Cohesiveness**	**Springiness**	**Gumminess**
Formulation	1.378	9.992 *	2.324	19.161 *
Process step	22.117 *	24.221 *	20.916 *	51.126 *
Interaction	2.207 *	4.030 *	10.286 *	1.894 *

* Statistical significance ≥ 95% (*p*-values ≤ 0.005). Values with different letters in the same row present significant differences.

## Data Availability

Raw data available on request to authors.
